# Relationships between pre-licensure academic performance and self-assessed clinical competence after graduation in recently graduated physical therapists

**DOI:** 10.20407/fmj.2025-029

**Published:** 2026-02-28

**Authors:** Tatsuya Kondo, Hiroaki Sakurai, Kei Ohtsuka, Nobushiro Okuchi, Yuta Ogawa, Hisanobu Hayashi, Yukari Suzuki, Yoshikiyo Kanada

**Affiliations:** 1 Graduate School of Health Sciences, Fujita Health University, Toyoake, Aichi, Japan; 2 Negotiation Office, Corporate Division, Semui Educational Institutions, Miyoshi, Aichi, Japan; 3 Faculty of Rehabilitation, School of Health Sciences, Fujita Health University, Toyoake, Aichi, Japan; 4 Department of Physical Therapy, Tokai Medical Science Academy, Nagoya, Aichi, Japan; 5 Department of Physical Therapy, Toyohashi Sozo University, Toyohashi, Aichi, Japan

**Keywords:** Pre-licensure academic performance, Clinical competence after graduation, CEPT

## Abstract

**Objectives:**

This study examined the relationship between pre-licensure academic performance and self-assessed clinical competence, as measured by the Clinical Competence Evaluation Scale in Physical Therapy (CEPT), among novice physical therapists with up to 1 year of clinical experience after graduation.

**Methods:**

The study participants were 37 graduates of a vocational school who had passed the national physical therapy licensure examination and were employed in healthcare or welfare facilities. Pre-graduation performance data included first-year and second-year academic grades, scores in basic and professional foundational subjects, and Clinical Training III evaluations. Self-assessments after graduation were conducted using the CEPT, which contains 53 items across seven competency categories. Correlational analyses were performed to investigate associations between pre-graduation academic metrics and CEPT scores.

**Results:**

There were significant negative correlations between first-year and second-year academic performance, scores in basic professional subjects, advanced professional subjects and the combined scores of basic and advanced professional subjects with CEPT domains related to “Knowledge” and “Decision-making skills.” Scores in basic professional subjects were the lowest among all categories and exhibited the strongest negative correlation with self-assessment.

**Conclusions:**

Students with higher levels of academic performance tended to rate themselves lower in early self-assessment after graduation, suggesting a possible lack of confidence in their knowledge or difficulties adapting to the complexities of clinical training. These findings highlight the potential value of integrating metacognitive support and self-assessment training into the physical therapy curriculum to foster practical competence and professional growth.

## Introduction

In recent years, ensuring a seamless transition from pre-licensure to clinical education after graduation has become a critical issue in physical therapy training. In Japan, revisions to national regulations in 2018 introduced structured assessments before and after clinical trainings, and mandates for visualizing learning outcomes.^1^ These reforms have helped establish a framework for evaluating students’ clinical competencies at the time of graduation. However, there is a lack of evidence regarding how these competencies evolve and how practitioners adapt in the early clinical environment after graduation.

Clinical competence in physical therapy encompasses a wide range of elements, including professional knowledge and skills, situational judgment, communication, ethical reasoning, and social adaptability.^2^ Although these abilities are initially cultivated during formal education, they are further refined through clinical experience and workplace-based learning. Therefore, clarifying how pre-licensure educational achievements influence clinical competence after graduation is essential for ensuring the quality of physical therapy education.

Particularly during the first year of clinical training, novice physical therapists undergo a transitional phase in which psychological and social adaptation is crucial. During this period, self-assessment plays a foundational role in helping therapists to recognize areas for improvement and set learning goals for professional development.^3^ Moreover, the accuracy of self-assessment has been shown to influence the development of clinical competence and self-efficacy.^4^

Early-stage professionals are reported to be susceptible to biased self-perceptions, including overestimation or underestimation of their competencies.^5^ Appropriate feedback and support from educational institutions and clinical supervisors are necessary to correct such discrepancies. Discrepancies between new graduates’ self-assessment and their institution’s evaluations may also indicate areas for improvement in educational assessment systems. Given this context, investigating the relationships between quantitative indicators of pre-graduation academic performance such as course grades, clinical training evaluations, and self-assessed clinical competence after graduation can yield valuable insights into educational programs and the design of continuous support systems. Previous reports indicate that academic performance during training can influence professional identity formation and self-efficacy after graduation.^6^ Although there have been some reports of correlations between clinical training evaluations and competence after graduation in physical therapy,^7^ comprehensive longitudinal investigations remain limited.

The current study investigated the relationships between pre-licensure academic performance—including grades in foundational, basic, and advanced professional subjects, and clinical training—and self-assessed clinical competence among physical therapists in their first year of professional practice.

## Methods

### Participants

The study participants were 52 graduates of a 3-year physical therapy program who had enrolled in 2019, passed the national licensure examination in 2022, and had begun working at hospitals or welfare facilities. After excluding individuals who had transferred from other institutions or left their initial place of employment within 1 year, 51 eligible graduates were contacted via mail. Thirty-seven individuals (16 men and 21 women; mean age at graduation: 23.7±3.0 years) responded and were included in the analysis. Their workplaces included hospitals (*n*=25), geriatric health facilities (*n*=2), outpatient clinics (*n*=6), and daycare centers (*n*=4). The study was approved by the Research Ethics Committee of Fujita Health University (HM22-341). Written informed consent was obtained by mail at the time of administering the Clinical Competence Evaluation Scale in Physical Therapy (CEPT), including permission to use participants’ pre-licensure academic performance for research purposes.

### Overview of the educational curriculum at our institution

The institution consists of eight departments; Nursing, Clinical Engineering, Physical Therapy, Occupational Therapy, Judo Therapy, Social Welfare, Mental Health Social Work, and Speech-Language-Hearing Sciences. The Physical Therapy department offers a 3-year curriculum composed of 11 foundational subjects, 18 basic professional subjects, and 28 advanced professional subjects.

In their first year, students complete 11 foundational, 13 basic professional, and five advanced professional subjects including Clinical training I. In their second year, students complete five basic professional subjects and 20 advanced professional subjects, including Clinical training II. In their third year, students complete three advanced professional subjects, including Clinical training III. Each year includes clinical placements: Clinical training I (one credit, 45 hours), which focuses on observational learning in hospitals; Clinical training II (three credits, 135 hours), which emphasizes assessment skills; and Clinical training III (16 credits, 720 hours), which emphasizes hands-on clinical skills and professional development. Clinical training III is conducted in two separate eight-credit placements at different facilities, with performance evaluations conducted for each.

### Pre-licensure education analysis

Analysis of pre-licensure education included course grades, clinical training evaluations, and national examination scores. Students scoring below 60 on regular examinations were retested and the higher score was recorded; however, in the current study only initial examination scores were used. The following averages were calculated: first-year grades, second-year grades (excluding Clinical trainings I and II), foundational subjects, basic professional subjects, advanced professional subjects (excluding all clinical trainings), the combined score of basic and advanced professional subjects, and the average Clinical training III score.

Figure 1 shows the evaluation form used in Clinical training III. Clinical training III evaluations included 42 items across five domains: Professional attitude and behavior (nine items), Basic knowledge of physical therapy (seven items), Attitude toward clinical training (six items), Physical therapy assessment skills (15 items), and Problem identification, treatment planning, and execution (five items). Each item was rated on a five-point scale, as follows:

A=Performs independently with minimal guidance

B=Performs appropriately with minimal guidance

C=Performs at an acceptable level with occasional guidance

D=Performs with significant support but below desired standards

E=Unable to perform even with full guidance.

Scores were calculated by first counting the number of responses rated A through E, assigning coefficients of 4 points for A, 3 points for B, 2 points for C, 1 point for D, and 0 points for E. The weighted sum was then divided by the total number of items, and the resulting value was computed for each of the two clinical training periods and combined. This combined value was subsequently converted to a scale where a maximum score of 4.0 corresponds to 100 points and a score of 2.0 corresponds to 60 points.

### Self-assessment of clinical competence after graduation

The CEPT^8^ was used to assess clinical competence after graduation (Table 1). The CEPT comprises 53 items across seven domains: “Knowledge of clinical physical therapy (Knowledge),” “Decision-making skills,” “Clinical skills,” “Communication skills,” “Attitude of health professionals,” “Self-learning abilities,” and “Self-management.”

Each item is scored on the following 4-point scale:

1=A large amount of instruction and advice needed;

2=Some instruction and advice needed;

3=Instructions from tutors are unnecessary, ability to perform as an autonomous practitioner;

4=Instructions from tutors are unnecessary, possesses a high level of competence to serve as a good example to other novice physical therapists and physical therapy students.

The total possible score is 212 points. The CEPT has demonstrated moderate to high intra-rater reliability as well as content, construct, and factorial validity when administered by a consistent evaluator. The CEPT was administered approximately 1 year after graduation. Participants were contacted by mail, and responses were collected via Google Forms, accessed by a URL provided in the enclosed document.

### Data analysis and statistical methods

To investigate relationships between pre-licensure academic performance and self-assessed competence after graduation, correlation analyses were conducted between CEPT domain/total scores and numerous variables, including first-year grades, second-year grades, foundational subjects, basic professional subjects, advanced professional subjects, combined basic and advanced professional subjects, and Clinical training III evaluations. Spearman’s rank correlation coefficients were calculated using IBM SPSS Statistics version 29.02 (IBM, Armonk, NY, USA), with a significance level set at *p*<0.05.

## Results

**Figure 2:**
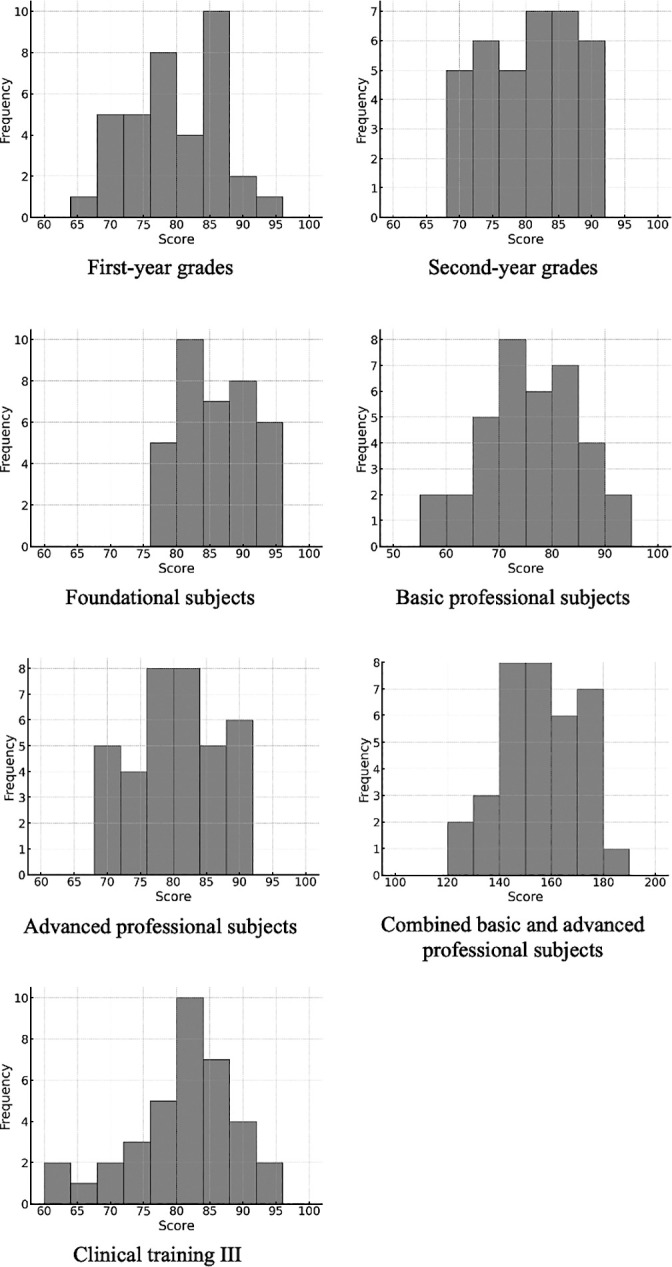
Histogram of pre-licensure academic performance

A histogram of pre-licensure academic performance is shown in Figure 2.

Pre-licensure academic performance means and standard deviations of academic scores included first-year grades: 79.3±7.1, second-year grades 80.0±6.7, foundational subjects 86.3±5.1, basic professional subjects 75.6±9.3, advanced professional subjects 80.0±6.6, combined basic and advanced professional subjects 147.4±37.9, and Clinical training III 80.4±7.7.

Mean CEPT domain scores and standard deviations were as follows: “Knowledge” 11.3±3.1, “Decision-making skills” 25.6±6.5, “Technical skills” 30.2±7.0, “Communication skills” 16.6±2.8, “Attitude of health professionals” 37.4±5.4, “Self-learning abilities” 11.3±2.2, “Self-Management” 11.5±2.2, and total CEPT score 132.6±22.1.

Table 2 shows the results of correlation analyses between pre-graduation education and the CEPT. There were significant negative correlations between first-year grades and “Knowledge” (rs –0.360, *p*=0.029), first-year grades and “Decision-making skills” (rs –0.371, *p*=0.024), second-year grades and “Decision-making skills” (rs –0.355, *p*=0.031), basic professional subjects and “Knowledge” (rs –0.356, *p*=0.030), basic professional subjects and “Decision-making skills” (rs –0.382, *p*=0.020), advanced professional subjects and “Decision-making skills” (rs –0.335, *p*=0.043), combined basic and advanced professional subjects and “Knowledge” (rs –0.335, *p*=0.050), and combined basic and advanced professional subjects and “Decision-making skills” (rs –0.378, *p*=0.021).

## Discussion

The results of the current study revealed significant negative correlations between higher pre-licensure academic performance in the first and second years, as well as in combined scores for basic professional subject and basic and advanced professional subjects, and lower CEPT scores in the “Knowledge” and “Decision-making skills” domains. These findings suggest that individuals with stronger academic records may tend to self-evaluate more conservatively in early clinical settings.

In a previous study, Yoshino^9^ administered the CEPT to first-year physical therapists with a duration of experience that was comparable to the participants in the present study and reported a median total score of 133 points (“Knowledge” 10 points, “Decision-making skills” 24 points, “Clinical skills” 28 points, “Communication skills” 16 points, “Attitude of health professionals” 34 points, “Self-learning abilities” 10 points, and “Self-management” 10 points). Although slight differences were observed across individual domains, the total score was generally consistent with that of the present study.^9^ These findings indicate that the CEPT results of the participants in this study were not atypical and can be considered comparable and valid relative to previous reports.

Eva et al.^3^ reported that self-assessments among early-stage professionals are often prone to bias, either through overestimation or underestimation. Another previous study reported that individuals with higher levels of cognitive abilities and self-monitoring capacity tend to be more critical of their own performance.^10^ High-performing students in the current study, when confronted with the complexities and uncertainties of real-world clinical training, may have become acutely aware of their own limitations in knowledge and decision-making, leading to lower self-assessment scores. In contrast, students in the low-performing group may have been unable to recognize or fully comprehend the complex issues and uncertainties encountered in clinical settings, which could have led them to assign higher self-assessment scores.

The CEPT, developed by Yoshino et al.,^8^ is designed to assess multidimensional clinical competence. This scale places greater emphasis on contextual knowledge application, interpersonal communication, and self-directed learning than the quantity of knowledge alone. This aligns with the observation in the current study that academic achievement did not directly correspond to higher CEPT scores. Furthermore, Yoshino^9^ reported that clinical competence in physical therapists steadily improved with experience, and that competence immediately after graduation was largely influenced by the amount of practical exposure the therapist had.^9^ Even students with a strong knowledge base may feel underprepared to fully utilize that knowledge during their early months of clinical work.

The relatively low performance in basic professional subjects is particularly noteworthy in the current study, which revealed the strongest negative correlations with CEPT domains related to “Knowledge” and “Decision-making skills.” This category includes essential biomedical disciplines such as anatomy, physiology, and general medicine, which constitute foundational knowledge that underpins clinical decision-making and intervention planning.^11^ In clinical settings, physical therapists must continuously apply this medical knowledge to accurately interpret symptoms and assess therapeutic risks. A lack of confidence in these areas may heighten anxiety and result in lower self-assessment in the early stages of practice.

High-performing students often excel in structured academic environments where there are clearly defined “right answers.” In contrast, clinical training demands decision-making in ambiguous, multifaceted situations involving interprofessional collaboration and patient communication; areas that cannot be mastered solely through textbook learning. This mismatch between academic evaluation and clinical reality may contribute to decreased confidence and lower self-ratings.

Regehr and Eva^12^ described a “theory-practice gap,” by which high academic achievers often struggle to adapt their theoretical knowledge to the unpredictable and context-dependent demands of clinical decision-making, particularly under conditions of uncertainty or incomplete information. Conversely, a subset of participants in the current study demonstrated relatively high self-assessment scores despite having lower academic performance. While confidence is an important aspect of clinical readiness, overestimation of one’s own competence in the absence of sufficient foundational knowledge or decision-making skills may carry the risk of inappropriate decision-making or reduced help-seeking behavior. This highlights the importance of fostering balanced self-perception, both for those who underestimate their abilities and those who may overrate their clinical readiness. From an educational standpoint, these findings underscore the necessity of promoting accurate and appropriately calibrated self-assessment behaviors from the early stages of professional training. Incorporating opportunities for structured reflection, comparison with peer and faculty feedback, and guided formative evaluation during pre-licensure education may assist students in developing a more realistic understanding of their competencies. Such efforts are essential to prevent both overconfidence and undue self-criticism, and to support safe, reflective, and autonomous practice after graduation.

The findings of the current study suggest that academic performance during pre-licensure education does not necessarily align with early self-assessed clinical competence, prompting reconsideration of how educational outcomes are evaluated and how feedback is delivered. Educational programs should incorporate instruction in self-assessment skills, and provide metacognitive support during clinical training.^13^

The current study involved several limitations that should be considered. First, the sample size was small and participants were recruited from a single institution, potentially limiting the generalizability of the findings. Second, although the CEPT has been validated and shown to be reliable, it remains a subjective measure of competence and may not perfectly reflect actual clinical performance. Third, because the study used a cross-sectional design, future longitudinal studies will be needed to track changes in self-assessment over time and evaluate their dynamic relationships with educational outcomes.

Moving forward, educational strategies should aim to bridge the gap between academic achievement and clinical competence. This may include enhancing early clinical experiences, fostering reflection and metacognitive skills, and explicitly cultivating self-assessment abilities as part of the curriculum. A potentially promising approach involves extending the duration of first-year clinical placements, thereby increasing opportunities for practical experience and structured reflection—both of which may contribute to addressing this issue. In addition, future investigations should explore the relationships between CEPT domains and disaggregated clinical training scores at the item level. Such analyses would help identify which specific aspects of clinical training most significantly contribute to the development of clinical competence and self-perception. These findings could provide concrete guidance for optimizing instructional content and prioritizing areas of focus within clinical education. Furthermore, there is a pressing need to standardize objective assessments of clinical competence and to establish educational approaches that incorporate both self-assessment and external evaluation.

## Figures and Tables

**Figure 1-1:**
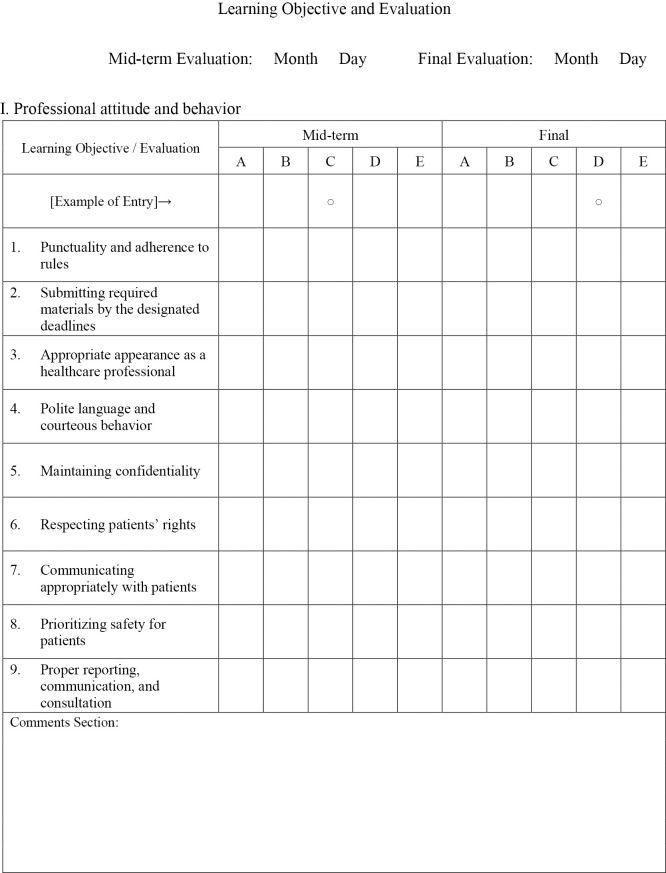
Professional attitude and behavior

**Figure 1-2:**
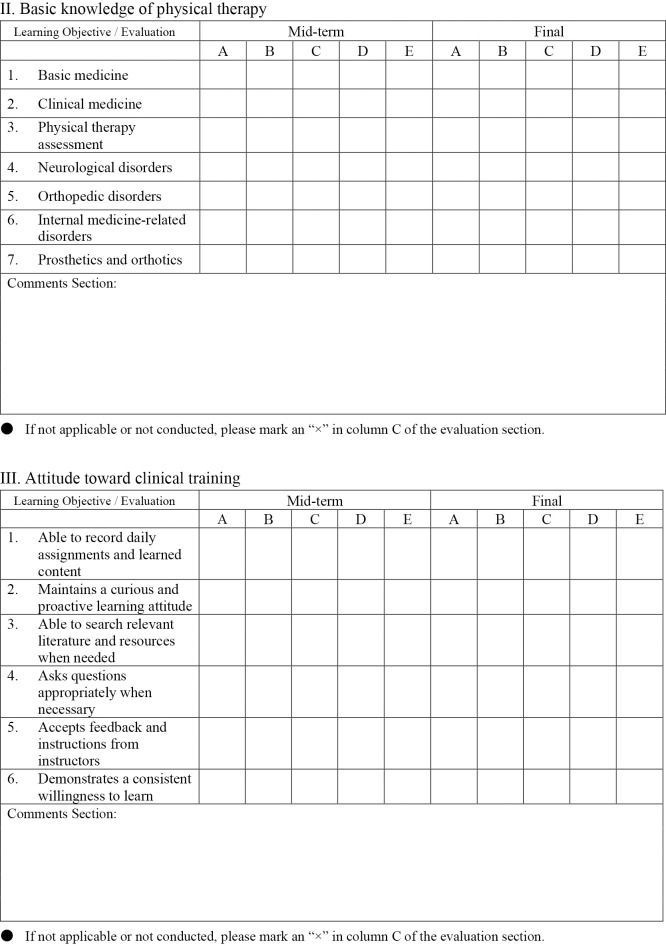
Basic knowledge of physical therapy and attitude toward clinical training

**Figure 1-3:**
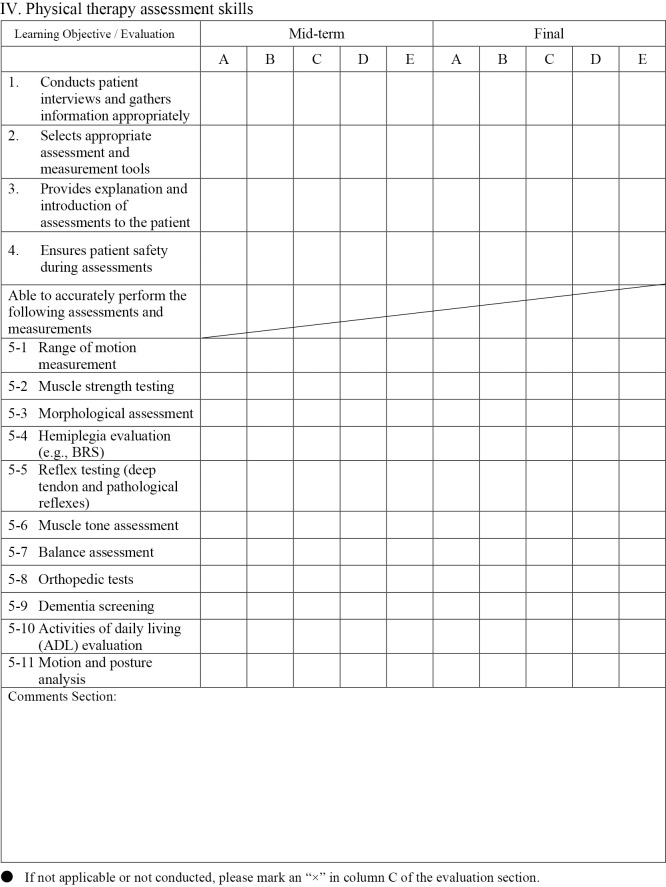
Physical therapy assessment skills

**Figure 1-4:**
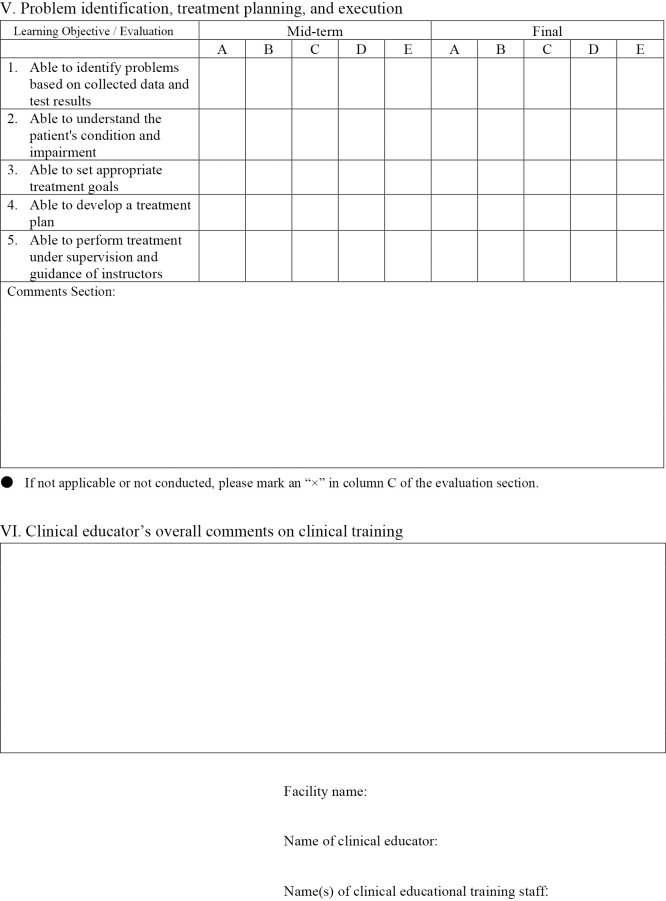
Problem identification, treatment planning and execution

**Table 1:** Overview of the Clinical Competence Evaluation Scale in Physical Therapy

Knowledge of clinical physical therapy	Understanding basic medical sciences, such as anatomy, physiology, and kinesiology Understanding nervous system diseases, such as cerebrovascular disorders, and neurodegenerative disease Understanding orthopedics, such as fracture, degenerative joint disease, and spinal cord injury Understanding internal medicine, such as cardiovascular, pulmonary disease, and metabolic disease Understanding medical and nursing-care insurance systems
Decision-making skills	Designing treatment plans by understanding the needs of clients and their families Designing treatment plans by understanding the progress, complications, medications, levels of bed rest, etc. Designing treatment plans by understanding social background, mental status, etc. Integration, interpretation, and identification of problems in symptoms, disabilities, and results of tests and measurements Understanding the client’s stage of disease (acute, convalescent, chronic) and designing appropriate treatment plans Noticing differences between the current client and the standard client Designing treatment plans according to the client’s progress and prognosis Designing various treatments according to the client’s symptoms and disabilities Treating while thinking about the effects of each treatment Determining therapeutic effects
Clinical skills	Choosing valid and reliable treatment measures Carrying out tests and measurements efficiently without burdening clients Proper contacting and touching clients without inducing anxiety or pain Having effective therapeutic skills Providing guidance to facilitate behavior modification in clients Providing safety guidance to comfortable assistance techniques to other health professionals and client’s family Writing well-organized medical records that are easy to understand Obtaining the latest knowledge through literature searches Accurately advising juniors or physical therapy students Treating and managing risk Dealing with an emergency and performing cardio-pulmonary resuscitation Dealing with complaints from clients, their families and other health professionals
Communication skills	Communicating with empathy according to the clients’ background and status Communicating to elicit the true needs of the clients and their family Explaining test results of tests measurement or treatment plans in an easy-to-understand manner Communicating with other health professionals and gathering information related to clients Presenting one’s ideas and opinions Listening and understanding the ideas and opinions of others
Attitudes of health professionals	Acting appropriately as a health professional Complying with the rules and manuals of the worksite Actively doing chores and improving the work environment Contacting and speaking to the client humbly Modifying incorrect behavior that has been pointed out Striving to help a difficult client to the end without giving up Treating clients with responsibility as the lead physical therapist Gaining the confidence of clients without rejection Gaining the confidence of colleagues and other health professionals Giving priority to and dedicating time to others Understanding and respecting the opinions of other health professionals Treating clients professionally
Self-learning abilities	Applying previous experience to current situations Continuing learning with ambition Actively asking senior or other health professionals questions Learning independently with specialty and interest
Self-management	Objectively analyzing one’s own behavior and acting with self-management Determining whether it is possible to complete a task by oneself and requesting help when necessary Understanding one’s role in an organization and acting in accordance with the role Managing one’s own physical condition and schedule and acting without disturbance on the job

**Table 2:** The results of correlation analyses between pre-licensure academic performance and the Clinical Competence Evaluation Scale in Physical Therapy

		Knowledge of clinical physical therapy	Decision-making skills	Clinical skills	Communication skills	Attitudes of health professionals	Self-learning abilities	Self-management	Total CEPT score
First-year grades	rs	–0.360*	–0.371*	–0.281	–0.159	–0.074	–0.183	–0.000	–0.272
	P-value	0.029	0.024	0.092	0.348	0.663	0.279	0.998	0.103
Second-year grades	rs	–0.309	–0.355*	–0.252	–0.181	–0.192	–0.085	–0.003	–0.266
	P-value	0.063	0.031	0.132	0.284	0.254	0.617	0.985	0.111
Foundational subjects	rs	–0.294	–0.254	–0.137	–0.044	0.092	–0.059	0.043	–0.143
	P-value	0.077	0.129	0.417	0.797	0.588	0.730	0.800	0.397
Basic pro.	rs	–0.356*	–0.382*	–0.292	–0.149	–0.116	–0.120	–0.029	–0.264
	P-value	0.030	0.020	0.079	0.380	0.495	0.481	0.866	0.115
Advanced pro.	rs	–0.261	–0.335*	–0.217	–0.163	–0.154	–0.061	0.046	–0.243
	P-value	0.119	0.043	0.196	0.334	0.362	0.721	0.785	0.148
Combined basic pro. and advanced pro.	rs	–0.335*	–0.378*	–0.284	–0.169	–0.156	–0.107	0.013	–0.278
	P-value	0.050	0.021	0.089	0.318	0.356	0.528	0.937	0.096
Clinical training III	rs	0.030	–0.001	–0.009	0.076	0.117	0.147	0.166	0.061
	P-value	0.860	0.995	0.957	0.656	0.490	0.386	0.326	0.721

*: *p*<0.05 Abbreviation: Basic pro., Basic professional subjects; Advanced pro., Advanced professional subjects.

## References

[1] Ministry of Health Labour and Welfare, Ministry of Education. Ministerial Ordinance on the Accreditation of Educational Institutions for Physical Therapists and Occupational Therapists; 2018 (in Japanese). <https://laws.e-gov.go.jp/law/341M50000180003?utm_source=chatgpt.com> (Accessed April 25, 2025)

[2] Furze JA, Black L, McDevitt AW, Kobal KL, Durning SJ, Jensen GM. Clinical reasoning: The missing core competency in physical therapist education and practice. Phys Ther 2022; 102: pzac093. 35781736 10.1093/ptj/pzac093

[3] Eva KW, Regehr G. Self-assessment in the health professions: a reformulation and research agenda. Acad Med 2005; 80: S46–S54. 16199457 10.1097/00001888-200510001-00015

[4] Fitzgerald JT, White CB, Gruppen LD. A longitudinal study of self-assessment accuracy. Med Educ 2003; 37: 645–649. 12834423 10.1046/j.1365-2923.2003.01567.x

[5] Govaerts MJ, Schuwirth LW, van der Vleuten CP, Muijtjens AM. Workplace-based assessment: effects of rater expertise. Adv Health Sci Educ Theory Pract 2011; 16: 151–165. 20882335 10.1007/s10459-010-9250-7 PMC3068251

[6] Chen Q, Zhang Q, Yu F, Hou B. Investigating structural relationships between professional identity, learning engagement, academic self-efficacy, and university support: Evidence from tourism students in China. Behav Sci 2023; 14: 26. 38247678 10.3390/bs14010026 PMC10813133

[7] Black JD, Palombaro KM, Dole RL. Student experiences in creating and launching a student-led physical therapy pro bono clinic: a qualitative investigation. Phys Ther 2013; 93: 637–648. 23431216 10.2522/ptj.20110430

[8] Yoshino J, Usuda S. The reliability and validity of the clinical competence evaluation scale in physical therapy. J Phys Ther Sci 2013; 25: 1621–1624. 24409034 10.1589/jpts.25.1621 PMC3885853

[9] Yoshino J. Study of the levels and secular changes of clinical competencies of physical therapysits. Rigakuryoho Kagaku 2021; 36: 699–704 (in Japanese).

[10] Govaerts MJ, van der Vleuten CP, Schuwirth LW, Muijtjens AM. Broadening perspectives on clinical performance assessment: rethinking the nature of in-training assessment. Adv Health Sci Educ Theory Pract 2007; 12: 239–260. 17096207 10.1007/s10459-006-9043-1

[11] Benbassat J, Baumal R, Borkan JM, Ber R. Overcoming barriers to teaching the behavioral and social sciences to medical students. Acad Med 2003; 78: 372–380. 12691966 10.1097/00001888-200304000-00009

[12] Regehr G, Eva K. Self-assessment, self-direction, and the self-regulating professional. Clin Orthop Relat Res 2006; 449: 34–38. 16735869 10.1097/01.blo.0000224027.85732.b2

[13] Sargeant J, Mann K, van der Vleuten C, Metsemakers J. “Directed” self-assessment: Practice and feedback within a social context. J Contin Educ Health Prof 2008; 28: 47–54. 18366127 10.1002/chp.155

